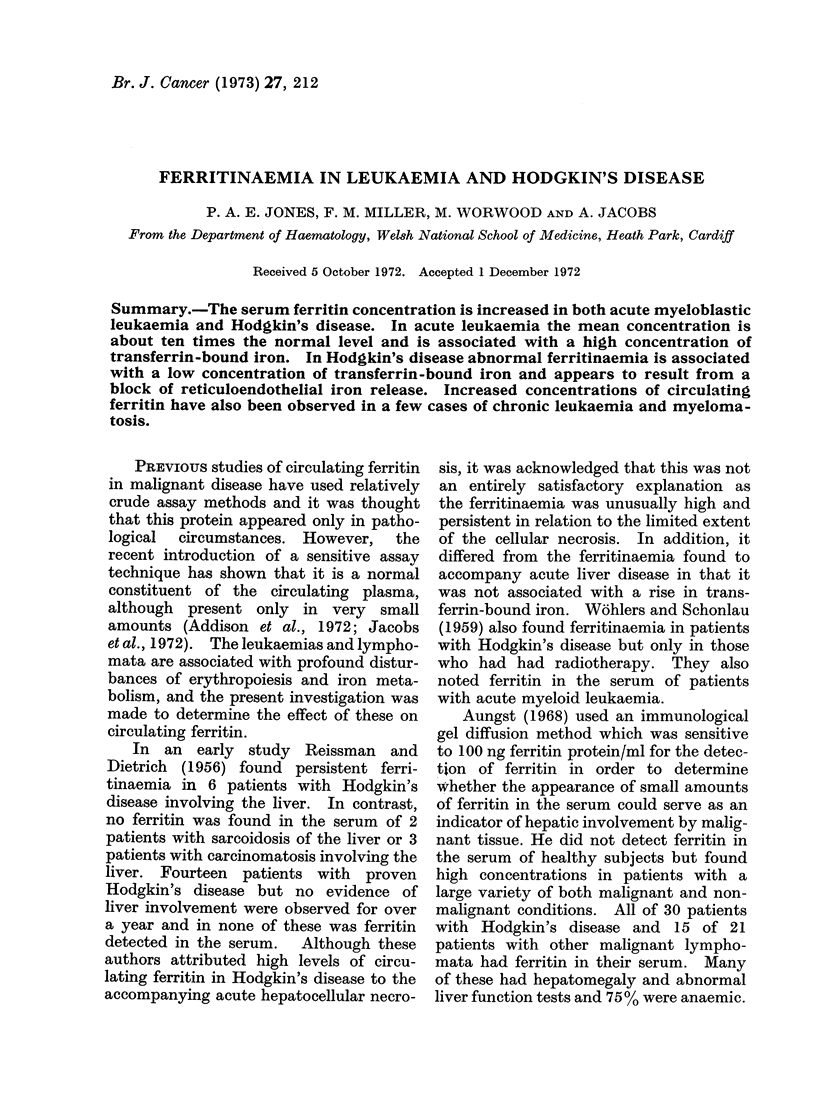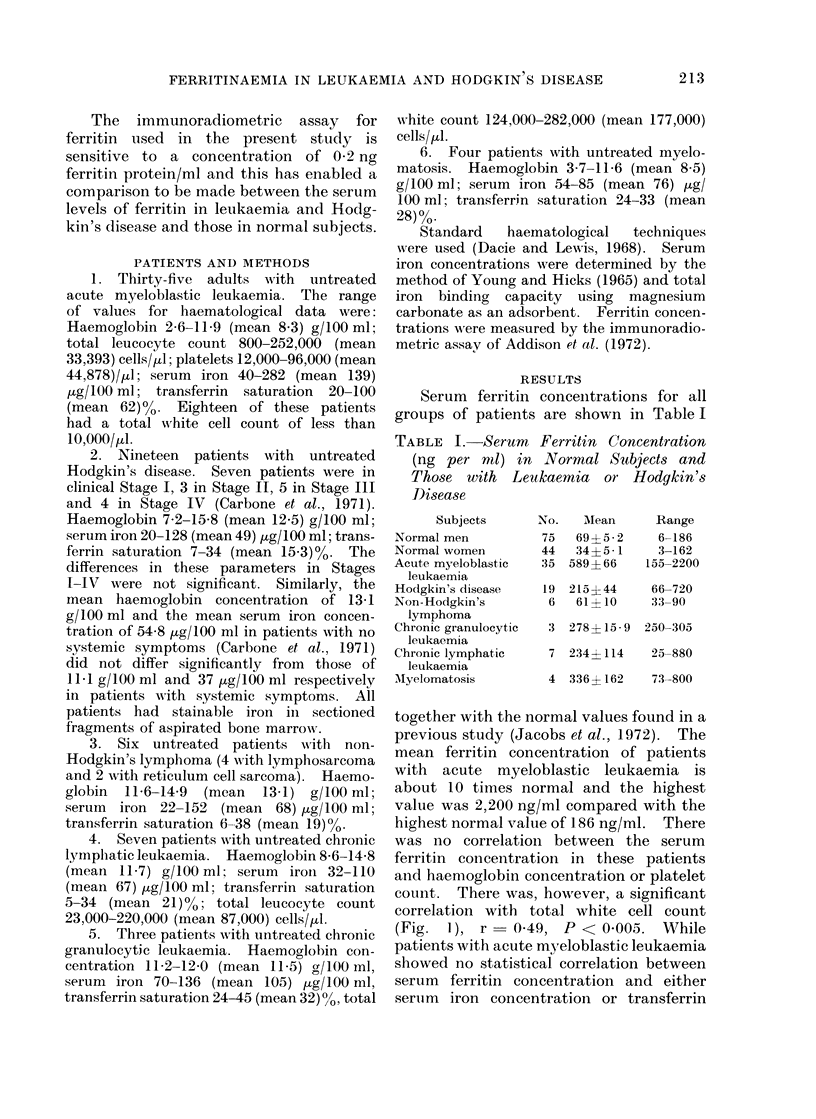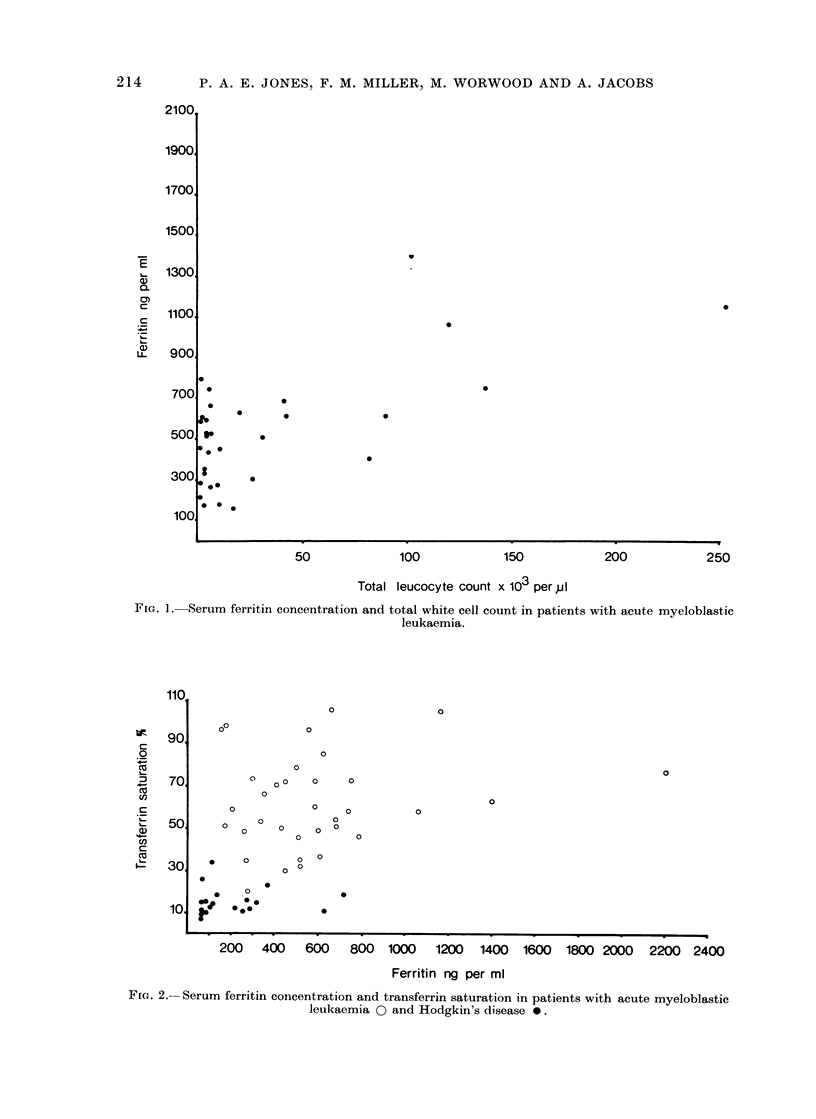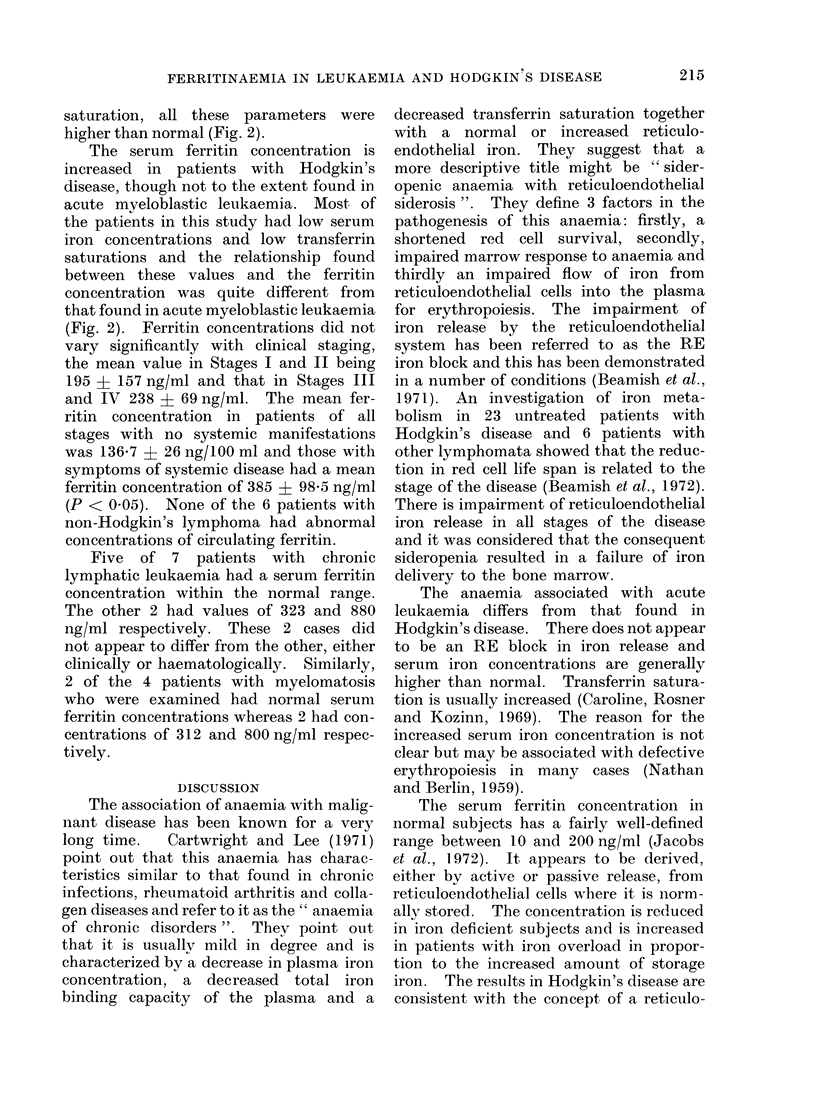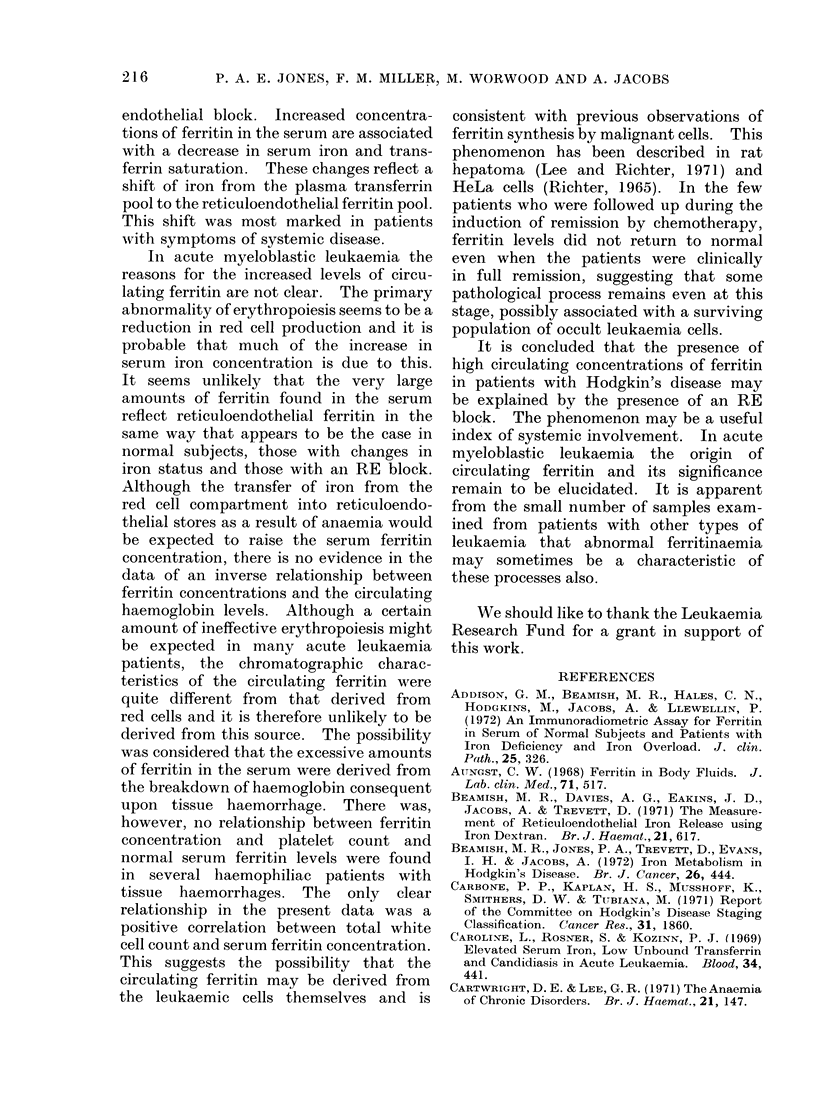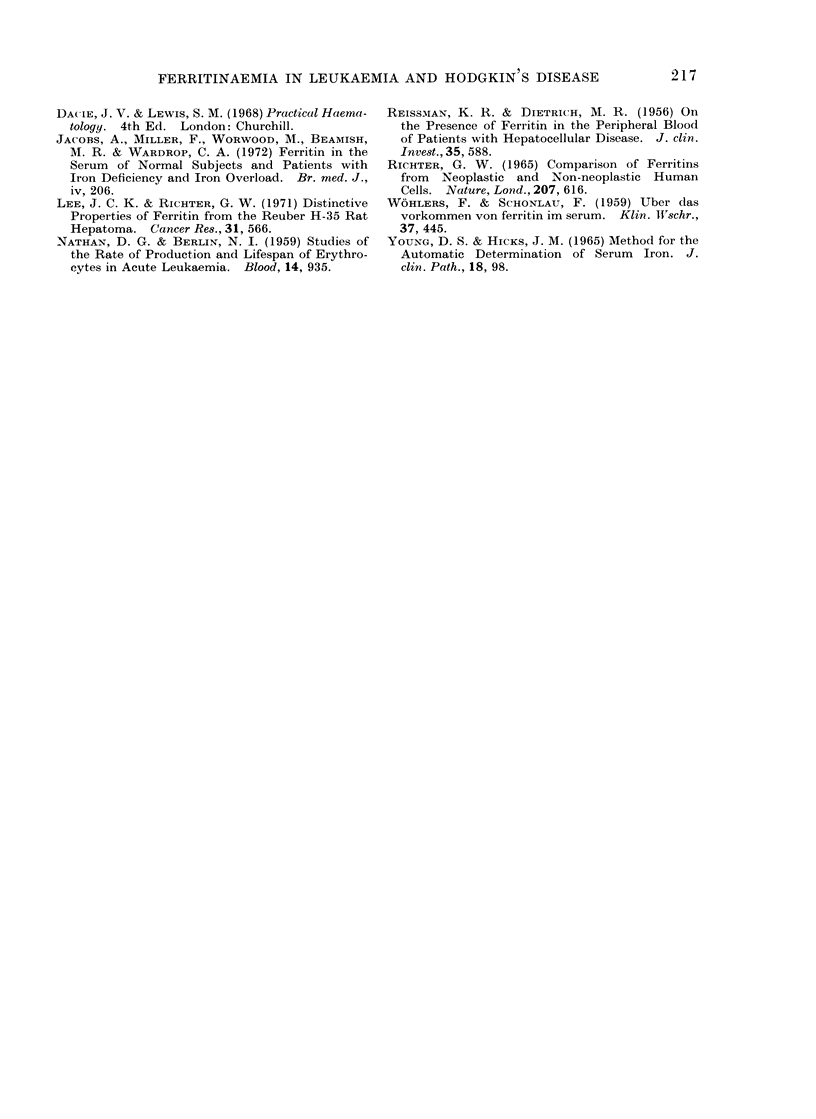# Ferritinaemia in Leukaemia and Hodgkin's Disease

**DOI:** 10.1038/bjc.1973.26

**Published:** 1973-03

**Authors:** P. A. E. Jones, F. M. Miller, M. Worwood, A. Jacobs

## Abstract

The serum ferritin concentration is increased in both acute myeloblastic leukaemia and Hodgkin's disease. In acute leukaemia the mean concentration is about ten times the normal level and is associated with a high concentration of transferrin-bound iron. In Hodgkin's disease abnormal ferritinaemia is associated with a low concentration of transferrin-bound iron and appears to result from a block of reticuloendothelial iron release. Increased concentrations of circulating ferritin have also been observed in a few cases of chronic leukaemia and myelomatosis.


*Br. J. Cancer* (1973) **27**, 212

### FERRITINAEMIA IN LEUKAEMIA AND HODGKIN'S DISEASE

P. A. E. JONES, F. M. MILLER, M. WORWOOD AND A. JACOBS

*From the Department of Haematology, Welsh National School of Medicine, Heath Park, Cardiff*

Received 5 October 1972. Accepted 1 December 1972

**Summary.—The serum ferritin concentration is increased in both acute myeloblastic leukaemia and Hodgkin's disease. In acute leukaemia the mean concentration is about ten times the normal level and is associated with a high concentration of transferrin-bound iron. In Hodgkin's disease abnormal ferritinaemia is associated with a low concentration of transferrin-bound iron and appears to result from a block of reticuloendothelial iron release. Increased concentrations of circulating ferritin have also been observed in a few cases of chronic leukaemia and myelomatosis.**

PREVIOUS studies of circulating ferritin in malignant disease have used relatively crude assay methods and it was thought that this protein appeared only in pathological circumstances. However, the recent introduction of a sensitive assay technique has shown that it is a normal constituent of the circulating plasma, although present only in very small amounts (Addison *et al.*, 1972; Jacobs *et al.*, 1972). The leukaemias and lymphomata are associated with profound disturbances of erythropoiesis and iron metabolism, and the present investigation was made to determine the effect of these on circulating ferritin.

In an early study Reissman and Dietrich (1956) found persistent ferritinaemia in 6 patients with Hodgkin's disease involving the liver. In contrast, no ferritin was found in the serum of 2 patients with sarcoidosis of the liver or 3 patients with carcinomatosis involving the liver. Fourteen patients with proven Hodgkin's disease but no evidence of liver involvement were observed for over a year and in none of these was ferritin detected in the serum. Although these authors attributed high levels of circulating ferritin in Hodgkin's disease to the accompanying acute hepatocellular necrosis, it was acknowledged that this was not an entirely satisfactory explanation as the ferritinaemia was unusually high and persistent in relation to the limited extent of the cellular necrosis. In addition, it differed from the ferritinaemia found to accompany acute liver disease in that it was not associated with a rise in transferrin-bound iron. Wöhlers and Schonlau (1959) also found ferritinaemia in patients with Hodgkin's disease but only in those who had had radiotherapy. They also noted ferritin in the serum of patients with acute myeloid leukaemia.

Aungst (1968) used an immunological gel diffusion method which was sensitive to 100 ng ferritin protein/ml for the detection of ferritin in order to determine whether the appearance of small amounts of ferritin in the serum could serve as an indicator of hepatic involvement by malignant tissue. He did not detect ferritin in the serum of healthy subjects but found high concentrations in patients with a large variety of both malignant and non-malignant conditions. All of 30 patients with Hodgkin's disease and 15 of 21 patients with other malignant lymphomata had ferritin in their serum. Many of these had hepatomegaly and abnormal liver function tests and 75% were anaemic.

The immunoradiometric assay for ferritin used in the present study is sensitive to a concentration of 0·2 ng ferritin protein/ml and this has enabled a comparison to be made between the serum levels of ferritin in leukaemia and Hodgkin's disease and those in normal subjects.

### PATIENTS AND METHODS

1. Thirty-five adults with untreated acute myeloblastic leukaemia. The range of values for haematological data were: Haemoglobin 2·6–11·9 (mean 8·3) g/100 ml; total leucocyte count 800–252,000 (mean 33,393) cells/μl; platelets 12,000–96,000 (mean 44,878)/μl; serum iron 40–282 (mean 139) μg/100 ml; transferrin saturation 20–100 (mean 62)%. Eighteen of these patients had a total white cell count of less than 10,000/μl.

2. Nineteen patients with untreated Hodgkin's disease. Seven patients were in clinical Stage I, 3 in Stage II, 5 in Stage III and 4 in Stage IV (Carbone *et al.*, 1971). Haemoglobin 7·2–15·8 (mean 12·5) g/100 ml; serum iron 20–128 (mean 49) μg/100 ml; transferrin saturation 7–34 (mean 15·3)%. The differences in these parameters in Stages I–IV were not significant. Similarly, the mean haemoglobin concentration of 13·1 g/100 ml and the mean serum iron concentration of 54·8 μg/100 ml in patients with no systemic symptoms (Carbone *et al.*, 1971) did not differ significantly from those of 11·1 g/100 ml and 37 μg/100 ml respectively in patients with systemic symptoms. All patients had stainable iron in sectioned fragments of aspirated bone marrow.

3. Six untreated patients with non-Hodgkin's lymphoma (4 with lymphosarcoma and 2 with reticulum cell sarcoma). Haemoglobin 11·6–14·9 (mean 13·1) g/100 ml; serum iron 22–152 (mean 68) μg/100 ml; transferrin saturation 6–38 (mean 19)%.

4. Seven patients with untreated chronic lymphatic leukaemia. Haemoglobin 8·6–14·8 (mean 11·7) g/100 ml; serum iron 32–110 (mean 67) μg/100 ml; transferrin saturation 5–34 (mean 21)%; total leucocyte count 23,000–220,000 (mean 87,000) cells/μl.

5. Three patients with untreated chronic granulocytic leukaemia. Haemoglobin concentration 11·2–12·0 (mean 11·5) g/100 ml, serum iron 70–136 (mean 105) μg/100 ml, transferrin saturation 24–45 (mean 32)%, total white count 124,000–282,000 (mean 177,000) cells/μl.

6. Four patients with untreated myelomatosis. Haemoglobin 3·7–11·6 (mean 8·5) g/100 ml; serum iron 54–85 (mean 76) μg/100 ml; transferrin saturation 24–33 (mean 28)%.

Standard haematological techniques were used (Dacie and Lewis, 1968). Serum iron concentrations were determined by the method of Young and Hicks (1965) and total iron binding capacity using magnesium carbonate as an adsorbent. Ferritin concentrations were measured by the immunoradiometric assay of Addison *et al.* (1972).

### RESULTS

Serum ferritin concentrations for all groups of patients are shown in Table I together with the normal values found in a previous study (Jacobs *et al.*, 1972). The mean ferritin concentration of patients with acute myeloblastic leukaemia is about 10 times normal and the highest value was 2,200 ng/ml compared with the highest normal value of 186 ng/ml. There was no correlation between the serum ferritin concentration in these patients and haemoglobin concentration or platelet count. There was, however, a significant correlation with total white cell count (Fig. 1), r = 0·49, $P < 0{\cdot}005$. While patients with acute myeloblastic leukaemia showed no statistical correlation between serum ferritin concentration and either serum iron concentration or transferrin

TABLE I.—*Serum Ferritin Concentration (ng per ml) in Normal Subjects and Those with Leukaemia or Hodgkin's Disease*

| Subjects | No. | Mean | Range |
|---|---|---|---|
| Normal men | 75 | 69±5·2 | 6–186 |
| Normal women | 44 | 34±5·1 | 3–162 |
| Acute myeloblastic leukaemia | 35 | 589±66 | 155–2200 |
| Hodgkin's disease | 19 | 215±44 | 66–720 |
| Non-Hodgkin's lymphoma | 6 | 61±10 | 33–90 |
| Chronic granulocytic leukaemia | 3 | 278±15·9 | 250–305 |
| Chronic lymphatic leukaemia | 7 | 234±114 | 25–880 |
| Myelomatosis | 4 | 336±162 | 73–800 |

FIG. 1.—Serum ferritin concentration and total white cell count in patients with acute myeloblastic leukaemia.

FIG. 2.—Serum ferritin concentration and transferrin saturation in patients with acute myeloblastic leukaemia ○ and Hodgkin's disease ●.

saturation, all these parameters were higher than normal (Fig. 2).

The serum ferritin concentration is increased in patients with Hodgkin's disease, though not to the extent found in acute myeloblastic leukaemia. Most of the patients in this study had low serum iron concentrations and low transferrin saturations and the relationship found between these values and the ferritin concentration was quite different from that found in acute myeloblastic leukaemia (Fig. 2). Ferritin concentrations did not vary significantly with clinical staging, the mean value in Stages I and II being $195 \pm 157$ ng/ml and that in Stages III and IV $238 \pm 69$ ng/ml. The mean ferritin concentration in patients of all stages with no systemic manifestations was $136{\cdot}7 \pm 26$ ng/100 ml and those with symptoms of systemic disease had a mean ferritin concentration of $385 \pm 98{\cdot}5$ ng/ml ($P < 0{\cdot}05$). None of the 6 patients with non-Hodgkin's lymphoma had abnormal concentrations of circulating ferritin.

Five of 7 patients with chronic lymphatic leukaemia had a serum ferritin concentration within the normal range. The other 2 had values of 323 and 880 ng/ml respectively. These 2 cases did not appear to differ from the other, either clinically or haematologically. Similarly, 2 of the 4 patients with myelomatosis who were examined had normal serum ferritin concentrations whereas 2 had concentrations of 312 and 800 ng/ml respectively.

### DISCUSSION

The association of anaemia with malignant disease has been known for a very long time. Cartwright and Lee (1971) point out that this anaemia has characteristics similar to that found in chronic infections, rheumatoid arthritis and collagen diseases and refer to it as the " anaemia of chronic disorders ". They point out that it is usually mild in degree and is characterized by a decrease in plasma iron concentration, a decreased total iron binding capacity of the plasma and a decreased transferrin saturation together with a normal or increased reticuloendothelial iron. They suggest that a more descriptive title might be " sideropenic anaemia with reticuloendothelial siderosis ". They define 3 factors in the pathogenesis of this anaemia: firstly, a shortened red cell survival, secondly, impaired marrow response to anaemia and thirdly an impaired flow of iron from reticuloendothelial cells into the plasma for erythropoiesis. The impairment of iron release by the reticuloendothelial system has been referred to as the RE iron block and this has been demonstrated in a number of conditions (Beamish *et al.*, 1971). An investigation of iron metabolism in 23 untreated patients with Hodgkin's disease and 6 patients with other lymphomata showed that the reduction in red cell life span is related to the stage of the disease (Beamish *et al.*, 1972). There is impairment of reticuloendothelial iron release in all stages of the disease and it was considered that the consequent sideropenia resulted in a failure of iron delivery to the bone marrow.

The anaemia associated with acute leukaemia differs from that found in Hodgkin's disease. There does not appear to be an RE block in iron release and serum iron concentrations are generally higher than normal. Transferrin saturation is usually increased (Caroline, Rosner and Kozinn, 1969). The reason for the increased serum iron concentration is not clear but may be associated with defective erythropoiesis in many cases (Nathan and Berlin, 1959).

The serum ferritin concentration in normal subjects has a fairly well-defined range between 10 and 200 ng/ml (Jacobs *et al.*, 1972). It appears to be derived, either by active or passive release, from reticuloendothelial cells where it is normally stored. The concentration is reduced in iron deficient subjects and is increased in patients with iron overload in proportion to the increased amount of storage iron. The results in Hodgkin's disease are consistent with the concept of a reticuloendothelial block. Increased concentrations of ferritin in the serum are associated with a decrease in serum iron and transferrin saturation. These changes reflect a shift of iron from the plasma transferrin pool to the reticuloendothelial ferritin pool. This shift was most marked in patients with symptoms of systemic disease.

In acute myeloblastic leukaemia the reasons for the increased levels of circulating ferritin are not clear. The primary abnormality of erythropoiesis seems to be a reduction in red cell production and it is probable that much of the increase in serum iron concentration is due to this. It seems unlikely that the very large amounts of ferritin found in the serum reflect reticuloendothelial ferritin in the same way that appears to be the case in normal subjects, those with changes in iron status and those with an RE block. Although the transfer of iron from the red cell compartment into reticuloendothelial stores as a result of anaemia would be expected to raise the serum ferritin concentration, there is no evidence in the data of an inverse relationship between ferritin concentrations and the circulating haemoglobin levels. Although a certain amount of ineffective erythropoiesis might be expected in many acute leukaemia patients, the chromatographic characteristics of the circulating ferritin were quite different from that derived from red cells and it is therefore unlikely to be derived from this source. The possibility was considered that the excessive amounts of ferritin in the serum were derived from the breakdown of haemoglobin consequent upon tissue haemorrhage. There was, however, no relationship between ferritin concentration and platelet count and normal serum ferritin levels were found in several haemophiliac patients with tissue haemorrhages. The only clear relationship in the present data was a positive correlation between total white cell count and serum ferritin concentration. This suggests the possibility that the circulating ferritin may be derived from the leukaemic cells themselves and is consistent with previous observations of ferritin synthesis by malignant cells. This phenomenon has been described in rat hepatoma (Lee and Richter, 1971) and HeLa cells (Richter, 1965). In the few patients who were followed up during the induction of remission by chemotherapy, ferritin levels did not return to normal even when the patients were clinically in full remission, suggesting that some pathological process remains even at this stage, possibly associated with a surviving population of occult leukaemia cells.

It is concluded that the presence of high circulating concentrations of ferritin in patients with Hodgkin's disease may be explained by the presence of an RE block. The phenomenon may be a useful index of systemic involvement. In acute myeloblastic leukaemia the origin of circulating ferritin and its significance remain to be elucidated. It is apparent from the small number of samples examined from patients with other types of leukaemia that abnormal ferritinaemia may sometimes be a characteristic of these processes also.

We should like to thank the Leukaemia Research Fund for a grant in support of this work.

REFERENCES

Addison, G. M., Beamish, M. R., Hales, C. N., Hodgkins, M., Jacobs, A. & Llewellin, P. (1972) An Immunoradiometric Assay for Ferritin in Serum of Normal Subjects and Patients with Iron Deficiency and Iron Overload. *J. clin. Path.*, **25**, 326.

Aungst, C. W. (1968) Ferritin in Body Fluids. *J. Lab. clin. Med.*, **71**, 517.

Beamish, M. R., Davies, A. G., Eakins, J. D., Jacobs, A. & Trevett, D. (1971) The Measurement of Reticuloendothelial Iron Release using Iron Dextran. *Br. J. Haemat.*, **21**, 617.

Beamish, M. R., Jones, P. A., Trevett, D., Evans, I. H. & Jacobs, A. (1972) Iron Metabolism in Hodgkin's Disease. *Br. J. Cancer*, **26**, 444.

Carbone, P. P., Kaplan, H. S., Musshoff, K., Smithers, D. W. & Tubiana, M. (1971) Report of the Committee on Hodgkin's Disease Staging Classification. *Cancer Res.*, **31**, 1860.

Caroline, L., Rosner, S. & Kozinn, P. J. (1969) Elevated Serum Iron, Low Unbound Transferrin and Candidiasis in Acute Leukaemia. *Blood*, **34**, 441.

Cartwright, D. E. & Lee, G. R. (1971) The Anaemia of Chronic Disorders. *Br. J. Haemat.*, **21**, 147.

DACIE, J. V. & LEWIS, S. M. (1968) *Practical Haematology*. 4th Ed. London: Churchill.

JACOBS, A., MILLER, F., WORWOOD, M., BEAMISH, M. R. & WARDROP, C. A. (1972) Ferritin in the Serum of Normal Subjects and Patients with Iron Deficiency and Iron Overload. *Br. med. J.*, iv, 206.

LEE, J. C. K. & RICHTER, G. W. (1971) Distinctive Properties of Ferritin from the Reuber H-35 Rat Hepatoma. *Cancer Res.*, **31**, 566.

NATHAN, D. G. & BERLIN, N. I. (1959) Studies of the Rate of Production and Lifespan of Erythrocytes in Acute Leukaemia. *Blood*, **14**, 935.

REISSMAN, K. R. & DIETRICH, M. R. (1956) On the Presence of Ferritin in the Peripheral Blood of Patients with Hepatocellular Disease. *J. clin. Invest.*, **35**, 588.

RICHTER, G. W. (1965) Comparison of Ferritins from Neoplastic and Non-neoplastic Human Cells. *Nature, Lond.*, **207**, 616.

WÖHLERS, F. & SCHONLAU, F. (1959) Uber das vorkommen von ferritin im serum. *Klin. Wschr.*, **37**, 445.

YOUNG, D. S. & HICKS, J. M. (1965) Method for the Automatic Determination of Serum Iron. *J. clin. Path.*, **18**, 98.